# PET/CT Radiomic Features: A Potential Biomarker for EGFR Mutation Status and Survival Outcome Prediction in NSCLC Patients Treated With TKIs

**DOI:** 10.3389/fonc.2022.894323

**Published:** 2022-06-21

**Authors:** Liping Yang, Panpan Xu, Mengyue Li, Menglu Wang, Mengye Peng, Ying Zhang, Tingting Wu, Wenjie Chu, Kezheng Wang, Hongxue Meng, Lingbo Zhang

**Affiliations:** ^1^ Positron Emission Tomography/Computed Tomography (PET-CT)/MR Department, Harbin Medical University Cancer Hospital, Harbin, China; ^2^ College of Bioinformatics Science and Technology, Harbin Medical University, Harbin, China; ^3^ Department of Pathology, Harbin Medical University Cancer Hospital, Harbin, China; ^4^ Oral Department, The Second Affiliated Hospital of Harbin Medical University, Harbin, China

**Keywords:** non-small cell lung cancer, PET-CT, radiomics, nomogram, EGFR mutation, survival prognosis

## Abstract

**Backgrounds:**

Epidermal growth factor receptor (EGFR) mutation profiles play a vital role in treatment strategy decisions for non–small cell lung cancer (NSCLC). The purpose of this study was to evaluate the predictive efficacy of baseline ^18^F-FDG PET/CT-based radiomics analysis for EGFR mutation status, mutation site, and the survival benefit of targeted therapy.

**Methods:**

A sum of 313 NSCLC patients with pre-treatment ^18^F-FDG PET/CT scans and genetic mutations detection were retrospectively studied. Clinical and PET metabolic parameters were incorporated into independent predictors of determining mutation status and mutation site. The dataset was randomly allocated into the training and the validation sets in a 7:3 ratio. Three-dimensional (3D) radiomics features were extracted from each PET- and CT-volume of interests (VOI) singularly, and then a radiomics signature (RS) associated with EGFR mutation profiles is built by feature selection. Three different prediction models based on support vector machine (SVM), decision tree (DT), and random forest (RF) classifiers were established. Furthermore, nomograms for estimation of overall survival (OS) and progression-free survival (PFS) were established by integrating PET/CT radiomics score (Rad-score), metabolic parameters, and clinical factors. Predictive performance was assessed by the receiver operating characteristic (ROC) analysis and the calibration curve analysis. The decision curve analysis (DCA) was applied to estimate and compare the clinical usefulness of nomograms.

**Results:**

Three hundred thirteen NSCLC patients were classified into a training set (n=218) and a validation set (n=95). Multivariate analysis demonstrated that SUVmax and sex were independent indicators of EGFR mutation status and mutation site. Eight CT-derived RS, six PET-derived RS, and two clinical factors were retained to develop integrated models, which exhibited excellent ability to distinguish between EGFR wild type (EGFR-WT), EGFR 19 mutation type (EGFR-19-MT), and EGFR 21 mutation type (EGFR-21-MT). The SVM model outperformed the RF model and the DT model, yielding training area under the curves (AUC) of EGFR-WT, EGFR-19-WT, and EGFR-21-WT, with 0.881, 0.851, and 0.849, respectively, and validation AUCs of 0.926, 0.805 and 0.859, respectively. For prediction of OS, the integrated nomogram is superior to the clinical nomogram and the radiomics nomogram, with C-indexes of 0.80 in the training set and 0.83 in the validation set, respectively.

**Conclusions:**

The PET/CT-based radiomics analysis might provide a novel approach to predict EGFR mutation status and mutation site in NSCLC patients and could serve as useful predictors for the patients’ survival outcome of targeted therapy in clinical practice.

## Introduction

Lung cancer is the leading cause of cancer-related death worldwide. Each year, approximately 1.6 million people die of lung cancer, and its five-year survival rate ranges from 4% to 17% (1). Histologically, non-small cell lung cancer (NSCLC) is the most frequent pathological subtype, which accounts for about 85% of the cases. Although early-stage lung cancer patients have a higher postoperative survival rate, treatments of advanced NSCLC show a relatively low response rate and significant toxicity (2). With the advance of precision medicine and personalized treatments, targeted therapy of NSCLC plays an increasingly important role as a rising star and was demonstrated to effectively improve the survival prognosis of lung adenocarcinoma patients with EGFR gene mutations (3). A series of previous studies (4, 5) have shown that patients with EGFR mutations exhibited longer overall survival (OS) and progression-free survival (PFS) than those with EGFR-WT when receiving tyrosine kinase inhibitors (TKIs) therapies. Additionally, regarding the most common sensitive mutations include exon 19 deletion (19DEL) and exon 21L858R, previous studies have demonstrated that patients with 19DEL mutations may have a greater survival benefit after TKIs treatment than those with 21L858R missing mutations (6, 7). Therefore, NSCLC therapies underwent an innovative transformation when it was realized that the mutant status of epidermal growth factor receptor (EGFR) directly affected the effectiveness of EGFR TKIs. It is critical to identify the molecular profiling of EGFR status in advanced NSCLC prior to individualized targeted therapy.

At present, clinical gene mutation detection usually uses tissue or cytological specimens, which has some disadvantages, such as trauma, difficulty in sampling, high cost, and unavoidable temporal and spatial heterogeneity of tumors (8). Analysis of circulating cell-free tumor DNA (ctDNA) is considered to be another emerging method for assessing EGFR mutation status (9). However, studies have shown that the ctDNA test has a relatively high false negative rate in clinical application, and the price is relatively high (10, 11). Therefore, there is an urgent need to develop noninvasive, simple, rapid, and reliable methods for gene mutation detection.

Radiomics is an emerging field in which a large number of quantitative imaging features are extracted from medical images to identify those most closely related to clinicopathologic, molecular, and genetic characteristics with the purpose of improving the diagnostic and prognostic accuracy (12). Although a series of works (13–15) have been reported to explore the potential relation between EGFR mutation status and radiomic features derived from CT images, only a few studies using PET/CT have been reported in this field. In the molecular imaging, it is often based on visual analysis or conventional parameters, maximum standardized uptake value (SUVmax), e.g., resulted in unideal predictive performance. Nevertheless, there is a lack of related researches integrating radiomics features with conventional semantic features. Moreover, previous studies mainly focused on the differentiation between EGFR-WT and EGFR-MT without involving the identification of specific mutation sites (EGFR-19-MT or EGFR-21-MT).

Therefore, the purpose of this study was to investigate whether radiomics features extracted from the same volume of interest (VOI) of PET and CT images combined with metabolic indexes and clinicopathological parameters could be used to predict EGFR mutation profiles and mutation site based on a tri-classification method. Furthermore, we intended to predict survival benefits of NSCLC patients treated with TKIs.

## Materials and Methods

### Patient Selection

**Figure 1:**
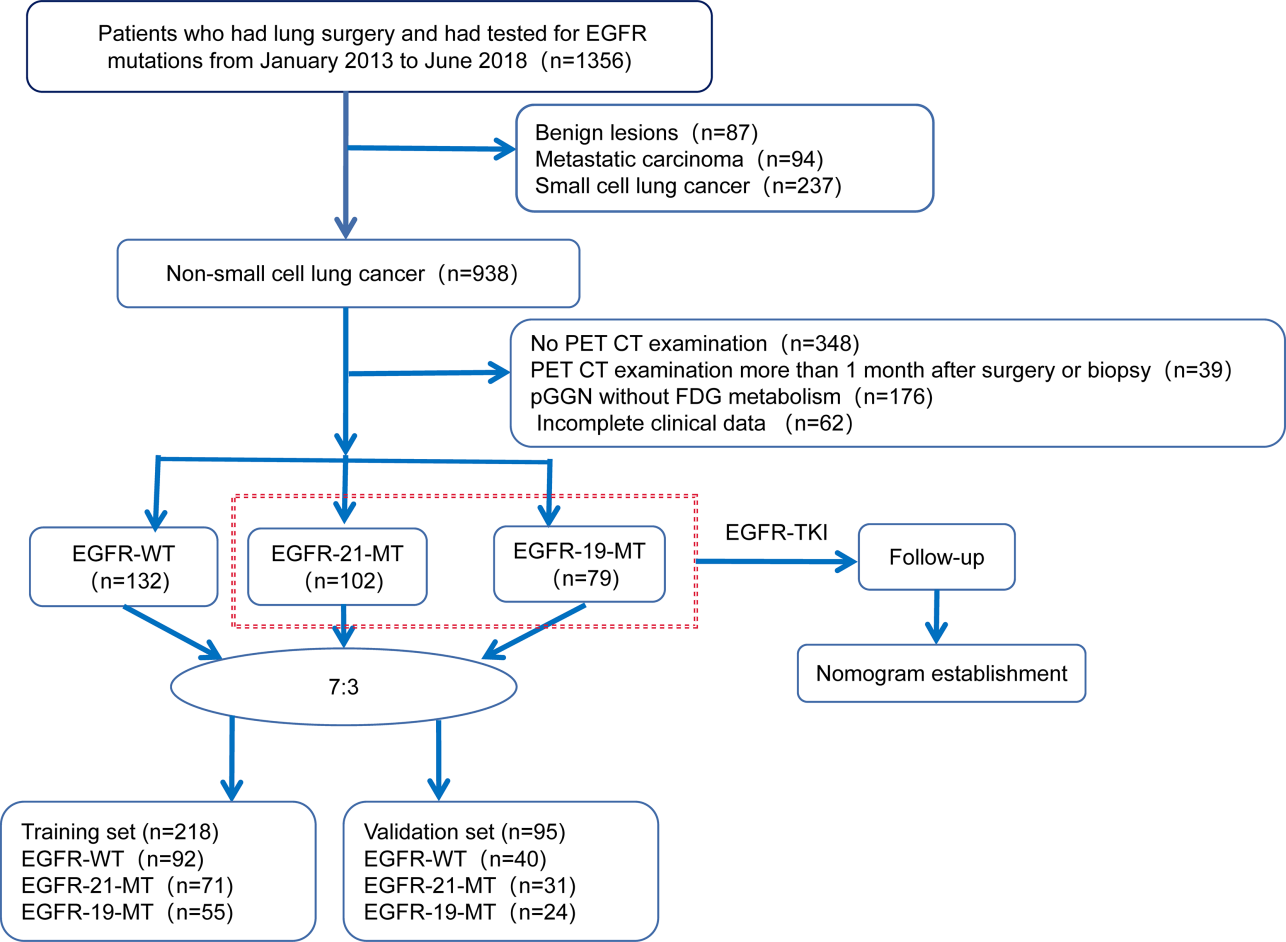
Study design and patient allocation.

This study was approved by the institutional review committee of Harbin Medical University Cancer Hospital. Given the retrospective nature of the study design and the anonymity of patient information, the informed consent requirement was waived. A total of 313 histologically proven NSCLC patients were retrospectively enrolled who underwent pretreatment ^18^F-FDG PET-CT scans in our hospital between January 2013 and June 2018. Inclusion criteria were as follows (1): pathologically confirmed NSCLC (2); PET-CT scans performed within one month prior to surgery or biopsy (3); no history of any antitumor therapy before scanning (4); no history of other malignancies (5); a single lesion with a maximum diameter ≥ 1 cm. Exclusion criteria were as follows (1): no genetic test for EGFR or unavailability of genetic test results (2) none or low FDG metabolism of pure ground-glass nodules (3) incomplete clinical data (4) difficulty in tumor margin delineation. Clinic-pathological information was obtained through clinical medical record retrieval, including age, gender, pathological stage, location, adenocarcinoma predominant subtype, carcinoembryonic antigen (CEA), smoking history and tumor size. Metabolic data including SUVmax, mean standardized uptake value (SUVmean) and total lesion glycolysis (TLG) were also recorded. The dataset was randomly assigned in a 7:3 ratio to the training cohort and validation cohort. Study design and patient allocation are shown in **Figure 1**. All cases in the training cohort were used to train the classification model, while cases in the validation cohorts were used to independently evaluate the model’s performance.

### EGFR Mutation Detection

Specific gene mutation information is confirmed by performing genetic testing on tumor tissue samples obtained by surgical resection or biopsy by an experienced physician. The mutation sites of four exons (exon 18-21) in the coding region of the EGFR gene were detected by real-time PCR. If any exon mutation was identified, the tumor was classified as EGFR-MT, otherwise considered as EGFR-WT.

### Image Acquisition

All patients fasted for more than 6 hours before scanning, and were tested blood glucose levels, which were kept below 11.0 mmol/L. The image acquisition was performed using the discovery VCT 64 PET/CT system (GE Healthcare, Milwaukee, USA). A 3.78 MBq/kg dose of FDG was administered intravenously. Approximate one hour later, whole-body CT scanning was performed with a standardized protocol consisting of 120 kV, 140 mA, and 3.75 mm slice thickness. Then, for PET, the images acquisition time was 2 minutes per bed position. Image reconstructions were performed based on the 3D ordered subset expectation-maximization algorithm (2 iterations and 17 subsets).

### Image Analysis, Tumor Segmentation and Radiomics Feature Extraction

The PET/CT images were analyzed by two radiologists blinded to the clinical and pathological results, (Reader 1, M.W and Reader 2, M.P with 15- and 20-years’ experience in the interpretation of PET/CT images, respectively). The metabolic parameters were measured by drawing a region-of-interest (ROI) on the axial PET image based on a threshold of 40% of SUVmax using commercial software (PET VCAR; GE Healthcare, USA). Any disagreement was resolved by consensus. SUVmax was defined at the highest value on one pixel with the highest counts within the ROI (16).

**Figure 2:**
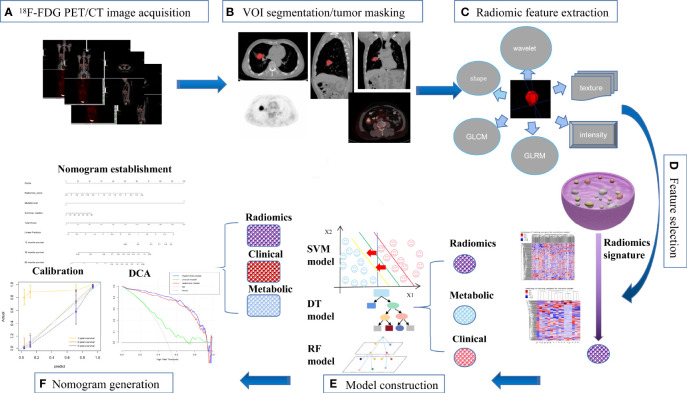
The workflow of our study. **(A)** image acquisition; **(B)** tumor masking; **(C)** feature extraction; **(D)** feature selection; **(E)** model construction; **(F)** nomogram generation.

The overview of radiomics workflow is displayed in **Figure 2**. Axial PET and CT digital imaging and communications in medicine images obtained from the picture archiving and communication system were applied for tumor segmentation. The tumor lesion was delineated separately on axial PET and CT images using LIFEx software (open-source software; www.lifexsoft.org/index.php). All 3D segmentation was first delineated automatically by means of a fixed threshold of 40% of the SUVmax, which were corrected by a radiologist manually afterward, blinded to surgical and pathological results.

We adopted three steps to preprocess the PET and CT images prior to feature extraction (17). Firstly, we resampled all images to a uniform voxel size of 1 mm × 1 mm × 1 mm using linear interpolation to minimize the influence of different layer thicknesses. Secondly, based on the gray-scale discretization process (bin width for CT = 25, bin width for PET = 0.1), we convert the continuous image into discrete values. Finally, we use the Laplacian of Gaussian and wavelet image filters to eliminate the mixed noise in the image digitization process in order to obtain low- or high-frequency features. Radiomics features were extracted from each PET-derived volume of interest (VOI) and CT-derived VOI by applying dedicated AK software (Artificial Intelligence Kit; GE Healthcare), which is in compliance with image biomarker standardization initiative guidelines (18). A total of 2074 radiomics features were extracted from each VOIs (1037 for CT, 1037 for PET) including (i) 198 for first-order feature, (ii) 14 for shape feature, (iii) 264 for gray level co-occurrence matrix (GLCM) feature, (iv) 176 for gray level size zone matrix (GLSZM) feature, (v) 176 for graGy level run length matrix (GLRLM) feature, (vi) 55 for neighborhood gray tone difference matrix (NGTDM) feature, (vii) 154 for gray level dependence matrix (GLDM) feature.

### Feature Selection

After the radiomics features extraction, Z-score normalization was done on each radiomics feature. In addition, the same preprocessing procedure was also applied to the testing set. The dataset was randomly assigned to either the training set or test set in 7:3 ratios. Intra- and inter-class correlation coefficients (ICCs) were calculated to assess the intra- and inter-observer reproducibility, and those radiomics signatures with ICC lower than 0.80 were excluded due to the poor reproducibility. Specifically, Reader 1 and Reader 2 drew the VOIs of 60 cases (20 EGFR-WT NSCLCs, 20 EGFR-19-MT NSCLCs and 20 EGFR-21-MT NSCLCs) of CT images and PET images randomly selected from the whole cohort. Reader 1 repeated the segmentations two weeks later. ICC greater than 0.80 indicated good agreement of feature extraction. The VOI segmentation for the remaining cases were performed by Reader 1.

The feature selection was carried out by using a stepwise selection method. Firstly, univariate logistic regression analysis was utilized to select features with *P* < 0.05 for the subsequent analysis. Secondly, multivariate logistic regression analysis was applied to choose features closely related to different EGFR status. The *P*-in and *P*-out of multivariate logistic analysis were 0.05 and 0.10, respectively. Finally, a subset of the most informative features was retained using the least absolute shrinkage and selection operator (LASSO) method.

### Machine Learning Model

Based on clinical variables, PET metabolic parameters, and PET/CT-derived radiomics features, three different machine learning classifiers were applied to develop a comprehensive model for differentiating between EGFR-WT, EGFR-19-MT, and EGFR-21-MT, respectively. A support vector machine (SVM) model was built bused on the selected optimal feature subsets of the training dataset. The hyper-parameters of the SVM model were automatically selected by the search method. The kernel, gamma and C were “rbf”, 0.1 and 0.1, respectively. Similarly, two other models using RF and DT classifiers were also established.

### Construction of Radiomics Nomograms

For patients receiving TKIs targeted therapy, all the clinical prognostic factors (including EGFR mutation site, gender, smoking status, pathological stage, location, histologic subtype, CEA, age and tumor size) and PET metabolic parameters (SUVmax, SUVmean and TLG) were evaluated by univariate analysis using the Kaplan-Meier approach. Statistically significant variables were analyzed for the multivariate Cox forward stepwise regression model to select independent predictors of OS and PFS. Cox regression models were utilized to select the most useful predictive features associated with patients’ survival outcomes. A PET/CT radiomics score (Rad-scores) was calculated for each patient by a linear combination of selected features weighted according to their respective coefficients, and corresponding nomograms were established by integrating the independent prognostic indicators as well as the Rad-score to assess survival benefit. To assess the clinical usefulness of the nomograms, C-index was calculated to evaluate the performance of the models, calibration curve analysis and DCA were performed for estimating and comparing the clinical usefulness of nomograms.

### Treatment, Follow Up and Survival Analysis

All patients with EGFR mutation type received first-line EGFR-TKI therapy and routine follow-up after treatment. The endpoints of this study were PFS and OS. PFS is defined as the time interval from treatment to recurrence or progression of the disease. OS is defined as the time interval from treatment to death. Survival curves were drawn using the Kaplan-Meier approach and compared using the log-rank test. Censored data were removed and all remaining data were used for survival analysis.

### Statistical Analysis

Univariate analysis (chi-square test or Mann-Whitney U test) was performed by using SPSS software (Version 25.0, IBM). The predictive performance of the machine learning models was determined by the receiver operating characteristic (ROC) curve, and area under the curve (AUC) were calculated. The “RMS” package was used to create the nomogram (19). All statistical analyses of this study were performed using R 3.5.1 and Python 3.5.6. A double-tailed *P* value less than 0.001 indicated statistical significance.

## Results

### Clinical Characteristics of Patients

A total of 313 NSCLC patients were enrolled in this study according to preset inclusion criteria, including 149 males and 164 females, with an average age of 59.21 ± 8.24 years (range 34–78). The sample included 102 cases of EGFR- 21-MT, 79 cases of EGFR-19-MT and 132 cases of EGFR-WT. The baseline information of all patients is displayed in **Table 1**. There were no significant differences between the training and validation sets in terms of age (*P* = 0.2244), TLG (Total Lesion Glycolysis) (*P* = 0.9373), tumor size (*P* = 0.0747), smoking history (*P* = 0.3849), pathological stage (*P* = 0.0675), tumor location (*P* = 0.4201) and carcinoembryonic antigen (CEA) level (*P* = 0.4076). Gender, Smoking history, SUVmax and SUVmean were significantly different between different EGFR mutation status in univariate logistic regression analysis in **Supplementary Table 1**. Multivariate logistic regression analysis revealed that only gender (EGFR-21-MT: OR =0.167, 95% CI [0.085-0.328], *P* < 0.001; EGFR-19-MT: OR =0.287, 95% CI [0.124-0.664], *P* < 0.001) and SUVmax (EGFR-21-MT: OR =1.186, 95% CI [1.122-1.253], *P* < 0.001; EGFR-19-MT: OR =1.330, 95% CI [1.241-1.424], *P* < 0.001) were independent predictors of EGFR mutation status and mutation site profiles in NSCLC patients in **Supplementary Table 2**.

**Table 1:** Demographic information and clinicopathological characteristics of selected patients with NSCLC.

Variable	EGFR-WT (n=132)	EGFR-21-MT (n=102)	EGFR-19-MT (n=79)	X²/Z	*p* value
**Sex**					
Female	40 (30.3)	75 (73.53)	49 (62.03)	47.0319	<0.001
Male	92 (69.7)	27 (26.47)	30 (37.97)		
**Age (years)**					
median	63.92 ± 8.71	62.62 ± 8.41	61.8 ± 9.9	1.5015	0.2244
**Pathological stage**					
I	0 (0)	0 (0)	0 (0)	8.7563	0.0675
II	4 (3.03)	10 (9.8)	11 (13.92)		
III	11 (13.92)	31 (30.39)	23 (29.11)		
IV	87 (65.91)	61 (59.8)	45 (56.96)		
**Location**					
Upper lobe	38 (28.79)	32 (31.37)	31 (39.24)	3.8972	0.4201
Middle lobe	43 (32.58)	34 (33.33)	18 (22.78)		
Lower lobe	51 (38.64)	36 (35.29)	30 (37.97)		
**Adenocarcinoma predominant subtype**					
Lepidic	12 (9.09)	14 (13.73)	24 (30.38)	4.2470	0.0557
Acinar	45 (34.09)	35 (34.31)	45 (56.96)		
Papillary	44 (33.33)	42 (41.18)	5 (6.33)		
Micropapillary	0 (0)	4 (3.92)	1 (1.27)		
Solid	31 (23.48)	7 (6.86)	4 (5.06)		
**CEA level**					
Normal (< 5 ng/ml)	84 (63.64)	60 (58.82)	43 (54.43)	1.7949	0.4076
Abnormal (≥ 5ng/ml)	48 (36.36)	42 (41.18)	36 (45.57)		
**Smoking history**					
Current or ever	49 (37.12)	34 (33.33)	22 (27.85)	1.9094	0.3849
Never	83 (62.88)	68 (66.67)	57 (72.15)		
**Tumor size (cm)**					
median	3 (2.4-3.2)	3 (2.1-3.2)	3.1 (2.66-3.45)	5.1873	0.0747
**SUVmax**					
median	16.91 (13.43-20.03)	24.75 (20.41-27.51)	28.62 (23.81-40.9)	113.5355	<0.001
**SUVmean**					
median	5.56 (4.46-8.4)	5.44 (4.42-7.04)	5.08 (4.12-6.69)	5.2287	0.0732
**TLG**					
median	284.43 (95.18-790.95)	344.67 (94.02-688.56)	270.98 (104.55-697.4)	0.1295	0.9373

SUVmax, maximum standardized uptake value; SUV, mean mean standardized uptake value; TLG, total lesion glycolysis; CEA, carcinoembryonic antigen.

### Survival Outcome

As of December 31, 2020, 163 of 181 populations had been successfully followed up regarding the OS and PFS in the nomograms-predicted set. The overall death rate was 48.47% (79/163) and the overall progression rate was 56.44% (92/163), respectively. The median OS of all populations was 25 months (range, 1-84 months), particularly 20 months (range, 1-59 months) for the EGFR-19-MT patients and 24 months (range, 2-84 months) for the EGFR-21-MT patients (log-rank test, *P* < 0.001). The median PFS of the patients was 21 months (range, 1-63months), particularly 16 months (range, 1-46 months) for the EGFR-19-MT patients and 20 months (range, 0.5-49 months) for the EGFR-21-MT patients (log-rank test, *P < 0.001).* The multivariate Cox regression analysis demonstrated that SUVmax and mutation site were independent prognostic indicators of both OS (HR=1.210 (95% CI) and 0.024 (95% CI), *P*< 0.001) and PFS (HR=1.001 (95% CI) and 0.026 (95% CI), *P* < 0.001). The corresponding survival curves were displayed in **Supplementary Figures 1–4**.

### Intra and Inter-Observer Reproducibility of Feature Extraction

The intra-observer ICC ranged from 0.809 to 0.914, and inter-observer ICC ranged from 0.758 to 0.900, therefore, an ideal intra- and inter-observer reproducibility of feature extraction was demonstrated in our study.

### Feature Extraction and Selection

A total of 2632 radiomics features were extracted from each VOIs (1316 for CT, 1316 for PET), and 14 radiomics features were filtered, which consisted of six CT-derived radiomics features and eight PET- derived radiomics features. The radiomic features and corresponding coefficients are listed in **Supplementary Table 3.**

### Performance of Different Prediction Models

**Figure 3:**
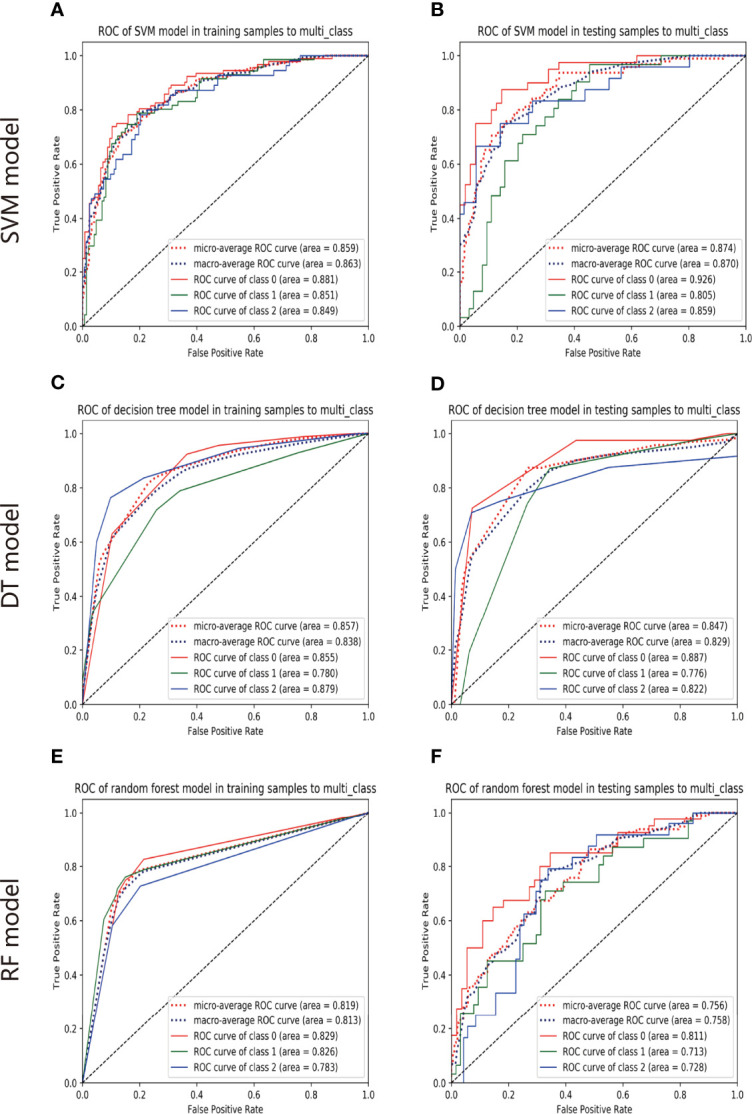
The predictive performance of models. The ROC of SVM model in training set **(A)** and validation set **(B)**. The ROC of DT model in training set **(C)** and validation set **(D)**. The ROC of RF model in training set **(E)** and validation set **(F)**.

The ROC analysis demonstrated clinical usefulness of the SVM model, which is superior to the DT model and RF model. All results regarding diagnostic efficacy were displayed in **Table 2** and the ROC curves were demonstrated in **Figure 3**. The AUC values of the SVM model in preoperative prediction of EGFR-WT, EGFR-21-MT and EGFR-19-MT were 0.881, 0.851 and 0.849, respectively in the training cohort, 0.926, 0.805, and 0.859, respectively in the validation cohort. The AUC values of the DT model in preoperative prediction of EGFR-WT, EGFR-21-MT and EGFR-19-MT were 0.881, 0.851 and 0.849, respectively in the training cohort, 0.926, 0.805 and 0.859, respectively in the validation cohort. The AUC values of the RF model in preoperative prediction of EGFR-WT, EGFR-21-MT and EGFR-19-MT were 0.881, 0.851 and 0.849, respectively in the training cohort, and 0.926, 0.805 and 0.859, respectively, in the validation cohort.

**Table 2:** The predictive performance (area under the curve) of three classifiers in Training set and Validation set.

Classifier	Training set			Validation set		
	EGFR-WT	EGFR-21-MT	EGFR-19-MT	EGFR-WT	EGFR-21-MT	EGFR-19-MT
**SVM**	0.881	0.851	0.849	0.926	0.805	0.859
**DT**	0.855	0.780	0.879	0.887	0.776	0.822
**RF**	0.829	0.826	0.783	0.811	0.713	0.728

### Construction and Validation of Radiomics Nomogram

Among clinical parameters, SUVmax and mutation sites proved to be independent predictors of OS and PFS, which was integrated into the nomogram’s development in **Supplementary Tables 4–7**. Radiomics features for calculating PET/CT Rad-scores of OS and their importance were displayed in **Table 3**. Radiomics features for calculating PET/CT Rad-scores of PFS and their importance were displayed in **Table 4**.

**Table 3:** Radiomic characteristics and significance of PET/CT radiomic scores (Rad-scores) used to calculate OS.

Feature name	Coefficient
original_glrlm_LowGrayLevelRunEmphasis.CT	7.7718
original_glszm_LowGrayLevelZoneEmphasis.CT	4.5851
wavelet.HHL_glcm_Correlation.CT	2.8216
wavelet.HLH_glszm_ZonePercentage.CT	0.2093
wavelet.LHH_gldm_SmallDependenceEmphasis.CT	-0.6163
original_shape_LeastAxisLength.PET	0.0040
wavelet.LHH_glcm_Idmn.PET	0.9181
wavelet.LLH firstorder Kurtosis.PET	0.0933

**Table 4:** Radiomic characteristics and significance of PET/CT radiomic scores (Rad-scores) used to calculate PFS.

Feature name	Coefficient
original_glrlm_ShortRunLowGrayLevelEmphasis.CT	9.9513
original_glszm_LowGrayLevelZoneEmphasis.CT	3.2857
original_shape_Flatness.CT	-0.4751
wavelet.HHL_glcm_Correlation.CT	1.4670
wavelet.LLH_glcm_Correlation.CT	1.3844
original_shape_LeastAxisLength.PET	0.0117
wavelet.HLL_glcm_Idn.PET	0. 1843
wavelet.LHH_glcm_Idmn.PET	0. 1424
wavelet.LLH firstorder Kurtosis.PET	0. 1105

**Figure 4:**
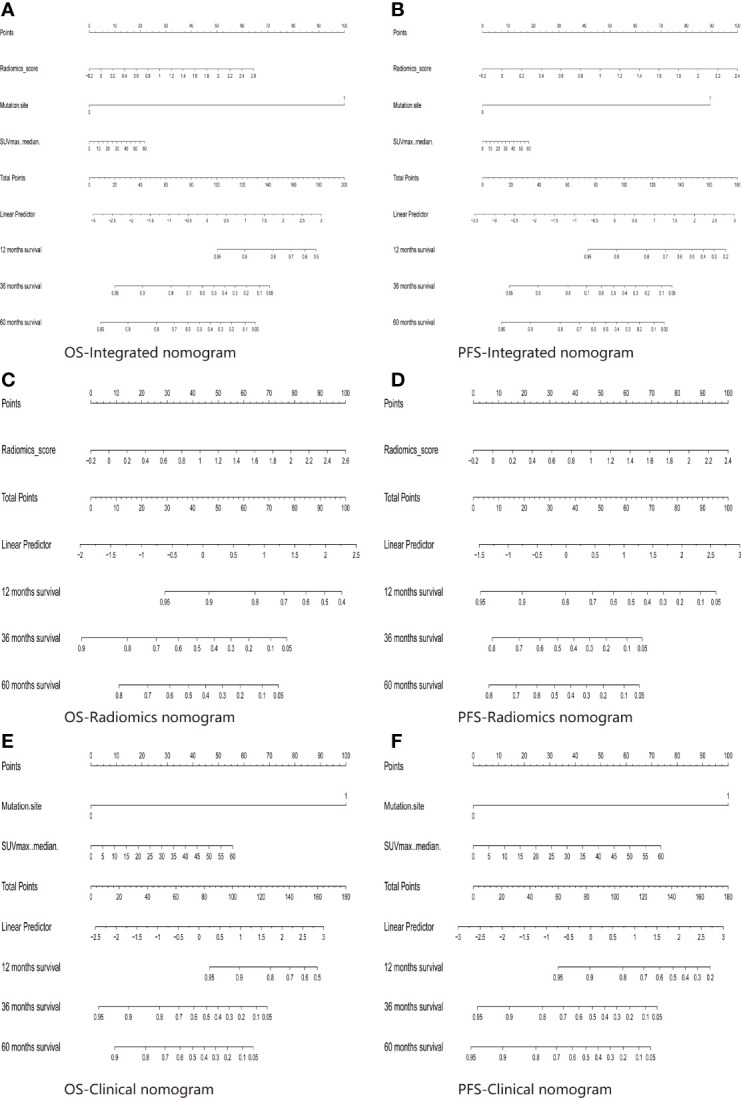
The Integrated model for OS **(A)** and PFS **(B)** prediction based on rad-score and clinical factors (mutation site, SUVmax). The Radiomics model for OS **(C)** and PFS **(D)** prediction based on rad-score. The Clinical model for OS **(E)** and PFS **(F)** prediction based on clinical factors (mutation site, SUVmax).

**Figure 5:**
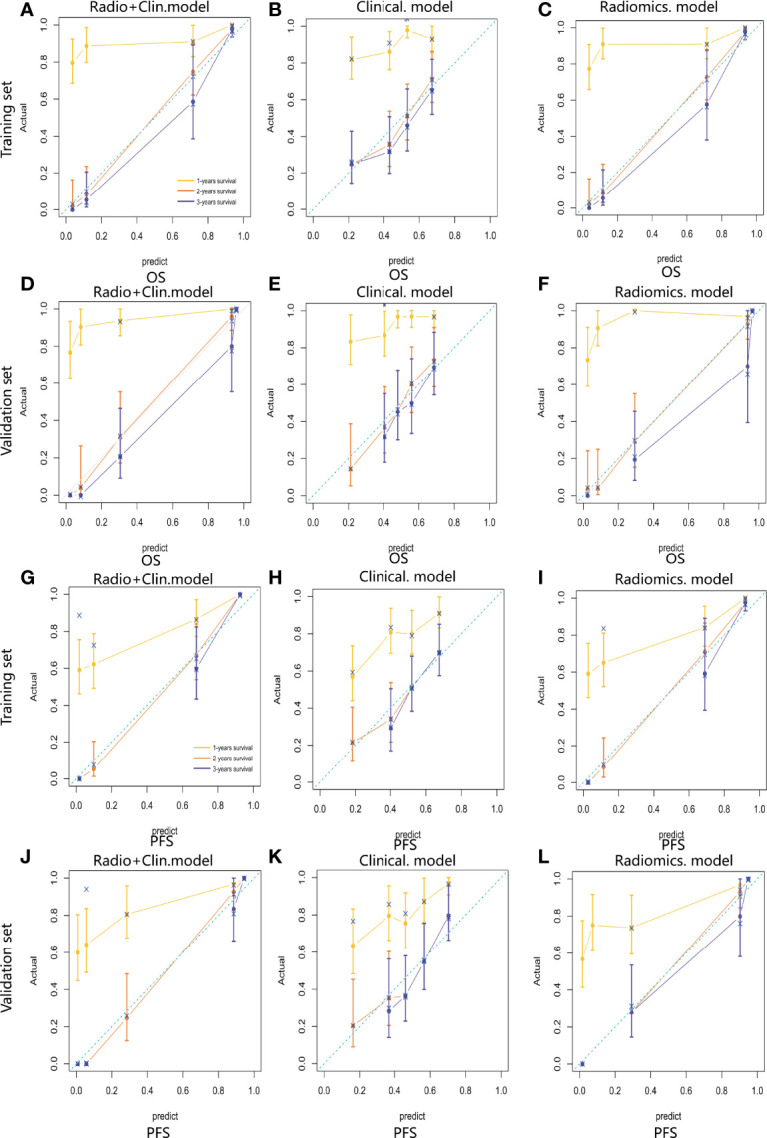
Calibration curve of the integrated model **(A)**, radiomics model **(B)** and clinical model **(C)** for OS estimation in the training set. Calibration curve of the integrated model **(D)**, radiomics model **(E)** and clinical model **(F)** for OS estimation in the validation set. Calibration curve of the integrated model **(G)**, radiomics model **(H)** and clinical model **(I)** for PFS estimation in the training set. Calibration curve of the integrated model **(J)**, radiomics model **(K)** and clinical model **(L)** for PFS estimation in the validation set.

**Figure 6:**
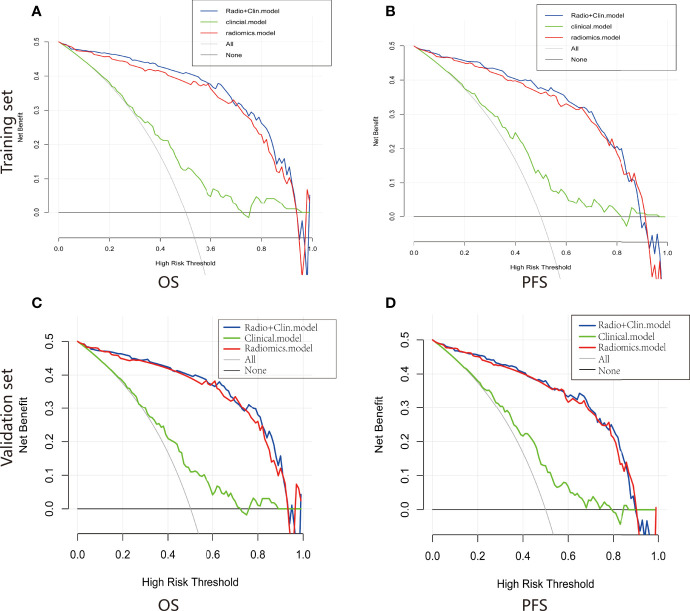
Decision curve of the nomograms for OS **(A)** and PFS **(B)** in the training set. Decision curve of the nomograms for OS **(C)** and PFS **(D)** in the validation set.

For estimation of OS, the C-indexs of the clinical nomogram in the training and validation sets were 0. 65 and 0.62, respectively. The C-index of the Integrated nomogram in the training set and validation set were 0.80 and 0.83, respectively. For estimation of PFS, the C-index of the clinical nomogram in the training and validation sets were 0.67 and 0.67, respectively. The C-index of the integrated nomogram in the training and validation sets were 0.80 and 0.82, respectively. The integrated nomogram outperformed the radiomics nomogram and the clinical nomogram. Nomograms were shown in **Figure 4**. The diagnostic performance of nomograms is shown in **Table 5**. The corresponding calibration curve and decision curve are displayed in **Figures 5**, **6**.

**Table 5:** Prognostic nomogram performance.

Model	OS				PFS			
	Training set		Validation set		Training set		Validation set	
	c-index	95% CI	c-index	95% CI	c-index	95% CI	c-index	95% CI
**Integrated nomogram**	0.80	0.75-0.84	0.83	0.78-0.87	0.80	0.75-0.85	0.82	0.78-0.87
**Radiomics nomogram**	0.80	0.75-0.84	0.82	0.77-0.86	0.79	0.74-0.84	0.82	0.77-0.86
**Clinical nomogram**	0.65	0.60-0.71	0.62	0.59-0.71	0.67	0.62-0.73	0.67	0.61-0.73

## Discussion

In summary, there are two highlights of our study. Firstly, we developed the first-of-its-kind PET/CT-derived radiomic signature based on the three-classification approach, which demonstrated excellent clinical usefulness in predicting EGFR mutation status. The radiomic signature successfully stratified NSCLC patients into EGFR-WT, EGFR-19-MT and EGFR-21-MT subgroups. Secondly, radiomics nomograms incorporating the radiomics signature were successfully established, demonstrating the incremental value of the radiomics signature to the conventional clinico-pathological factors for individualized survival estimation.

In this study, we firstly explored the potential association between PET metabolic parameters and the EGFR mutation profiles. Our findings demonstrated that there was a significant difference in SUVmax between EGFR-WT, -19-MT and -21-MT patients. Similarly, in a previous study conducted by Lv et al. (20) confirmed that ^18^F-FDG PET/CT metabolic parameters’ values were significantly lower in EGFR-MT than in EGFR-WT NSCLCs. Another previous study also reported that EGFR-MT lung adenocarcinomas have relatively lower ^18^F-FDG uptake in comparison with EGFR-WT tumors (21), and SUVmax of patients EGFR-21-MT was higher than that of EGFR-19-MT (22). The possible reasons are explained as follows: EGFR mutation was correlated with low tumor metabolic activity of NSCLCs on ^18^F-FDG PET/CT. Several researchers considered that EGFR-TKIs could accelerate the glucose uptake of tumor cells. Specifically, tumor cells with high glucose metabolism levels have abundant glucose uptake. Thus, they have less demand for EGFR-TKIs compared to low metabolic tumor cells. As a result, the incidence of EGFR-MT in NSCLCs with high SUVmax is relatively lower (23). Our results are in line with such conclusions. However, different from other acceptable notions, Results from Lee et al. (24) and Minamimoto et al. (25). Indicated that no significant difference was found regarding the SUVmax between the EGFR-WT and EGFR-MT patients, suggesting that SUVmax was not an independent predictor for EGFR mutation. Previous studies conducted by Kanmaza et al. (26) and Ko et al. (27) demonstrated that a higher SUVmax was associated with an EGFR mutation. As a result, these conflicting results demonstrated that ^18^F-FDG uptakes may not be a dependable marker for predicting EGFR mutation status. The possible reasons for these discrepant findings can be attributed by the patient baseline demographics of the enrolled patients, the small study sample size number of patients in our study, and the complex tumor microenvironment.

Although a significant relationship between the tumor glucose metabolism level depicted on PET images and EGFR mutation profiles has been reported in several works (22, 28, 29), traditional PET-derived semiquantitative indexes show insufficient ability to be widely used in clinical practice. It has been demonstrated that SUVmax as a single pixel value only yield moderate AUC for differentiating EGFR-WT from EGFR-MT, whereas TLG as a volumetric measurement of glucose metabolism level has not demonstrated more satisfactory performance either. Thus, our study established a comprehensive prediction model based on ^18^F-FDG PET/CT radiomics analysis to provide additional value in optimizing the predictive performance for EGFR mutation profiles in patients with NSCLC.

Radiomics, as an emerging field, has greatly promoted the diagnostic and prognostic accuracy. Currently, radiomics for determining gene mutation status in patients with NSCLC based on PET/CT images were reported in several studies (29–31). In a previous study, Zhang et al. (32) developed radiomics model to assess the predictive power of pre-therapy ^18^F-FDG PET/CT-based radiomic features for EGFR mutation status in NSCLC. However, firstly, it was carried out on a relatively small sample size (two hundred and forty-eight patients). Secondly, the area under the curve values analysis for predicting EGFR mutation status displayed limited discrimination performances (with AUC equal to 0.79 in the training set, and 0.85 in the validation set). Thirdly, advanced radiomics features were not extracted for all patients for technical reasons (only 47 PET and 45 CT radiomic features). In contrast, multiple machine learning classifiers were utilized to identify predictive radiomic features, and the SVM model yielded a training AUC of 0.881, 0.851 and 0.849 in EGFR-WT, EGFR-19-WT and EGFR-21-WT, respectively, whereas a validation AUC of 0.926, 0.805 and 0.859, respectively in the current study, which might provide higher diagnostic performance.

The current study was applied relatively larger sample size, higher-order features and advanced radiomics analysis methods, as well as high-dimensional radiomics signatures extracted up to 2632. Li et al. (33) developed radiomics model through an integrated analysis of 115 NSCLC patients with somatic mutation testing to investigate the feasibility of quantitative and qualitative features extracted from PET-CT in evaluating EGFR mutation status in NSCLC patients. Only a total of 38 radiomic features quantifying tumor morphological, grayscale statistic, and texture features were extracted from the primary PET/CT images. A radiomic signature based on both PET and CT radiomic features outperformed individual radiomic features, the PET or CT radiomic signature. Additionally, a combined radiomic signature with clinical factors exhibited a further improved performance in EGFR mutation status differentiation in NSCLC. In the present study, we also constructed the different classifiers based on integrated radiomic features derived from PET, CT and metabolic parameters to further improve the diagnostic ability.

Furthermore, in terms of predicting EGFR gene mutations in NSCLC, few studies involve predicting the certain EGFR mutation site (EGFR-19-MT or EGFR-21-MT) using PET-CT. The study of Zhang et al. (34) have validated that only one PET radiomics feature demonstrated significant but low predictive ability (AUC = 0.661) for differentiating EGFR-19-MT from EGFR-21-MT. Compared with the above study, our prediction model can distinguish EGFR-WT, EGFR-19-MT and EGFR-21-MT in one stop, and shows good discrimination performance.

Regarding strengths of the present work, our results not only predicted EGFR mutation status and mutation site, but also predicted patient survival outcomes, which have scarcely been investigated. In clinical practice, although the tumor, node, and metastasis (TNM) staging system are commonly applied to evaluate the survival prognosis of malignant tumors, we have to admit that this method still has many inevitable shortcomings in the prognostic assessment of lung cancer (35). In fact, the survival period of patients at the same stage may differ. Thus, a one-size-fits-all strategy based on TNM is not applicable in all situations. Novel methods of prognostic assessment are urgently needed to achieve precision treatment. In this study, we supplied clinicians with an easy-to-use method for predicting survival outcomes in NSCLC patients receiving targeted therapy by constructing a radiomics nomogram that exhibited excellent performance, with high c-indexes in the validation set. Furthermore, with the inclusion of clinic-pathological variables in a single nomogram, the prediction performance was further improved, which could allow for better decision-making for NSCLC patients. In the present study, we found that SUVmax and mutation site were independent predictors of the survival period, suggesting their clinical usefulness in the long-term management of NSCLC patients receiving TKIs. Our data provided concordant results to previous study that SUVmax can provide some evidence for survival prognosis (36). On the basis of this fact, we guess that the higher the level of glucose metabolism, the more aggressive tumor cell growth is, and the poorer the patient’s survival prognosis is (37). Yang et al. demonstrated that gender was an important prognostic risk factor in NSCLC patients receiving TKI therapy, which is inconsistent with our findings, possibly due to differences in the inclusion of the study population (38).

Although this study has obtained satisfactory results, there are still several limitations: Firstly, patient selection bias might exist due to the retrospective nature. Thus, a prospective validation might provide sufficient evidence for further clinical application. Secondly, cases from a single center and relatively small sample size may impair the portability of the prediction model. It is necessary to conduct multi-center research to enhance the generalization ability of the model. Thirdly, only lung adenocarcinoma was included in this study. The predictive ability of our model in other lung cancer types is needed to be validated. Fourth, as for the delineation of the lesions, a semi-automatic segmentation method is used. The more time-consuming approach should be explored in the future.

## Conclusion

In conclusion, this study demonstrated that the pre-treatment PET/CT-based radiomics features exhibited excellent performance for the prediction of EGFR mutation profiles in lung adenocarcinoma. Furthermore, we provided an easy-to-use approach to predict the survival outcome of patients receiving targeted therapy, which can be very useful in the clinical practice to guide individualized molecular targeted therapy.

## Data Availability Statement

The raw data supporting the conclusions of this article will be made available by the authors, without undue reservation.

## Ethics Statement

The studies involving human participants were reviewed and approved by Harbin Medical University Cancer Hospital. The ethics committee waived the requirement of written informed consent for participation.

## Author Contributions

Conception and design: LY and PX. Collection and assembly of the data: PX. Development of the methodology: ML and YZ. Data processing: MW and MP. Data analysis and interpretation: WC and TW. Manuscript writing: All authors. Manuscript review: LZ, HM, and KW. All authors contributed to the article and approved the submitted version.

## Funding

This paper is supported by the National Natural Science Foundation of China General Projects (81571740), Provincial Key Research and Development Program of Heilongjiang Province (GA21C001), Postdoctoral Special Scientific Research Grant of Heilongjiang Provincial Government (LBH-Q17104), Distinguished Young Scientist Funding of Harbin Medical University Affiliated Tumor Hospital (JCQN2019-02, Key Project of the Climbing Funding of the National Cancer Center (NCC201808B019), Haiyan Funding of Harbin Medical University Cancer Hospital (JJQN2019-23). The funders had no role in study design, data collection and analysis, decision to publish, or preparation of the manuscript.

## Conflict of Interest

The authors declare that the research was conducted in the absence of any commercial or financial relationships that could be construed as a potential conflict of interest.

## Publisher’s Note

All claims expressed in this article are solely those of the authors and do not necessarily represent those of their affiliated organizations, or those of the publisher, the editors and the reviewers. Any product that may be evaluated in this article, or claim that may be made by its manufacturer, is not guaranteed or endorsed by the publisher.

## Abbreviations

EGFR, Epidermal growth factor receptor; NSCLC, non–small cell lung cancer; TKI, tyrosine kinase inhibitor; PET, positron emission tomography; SUVmax, maximum standardized uptake value; SUVmean, mean standardized uptake value; TLG, total lesion glycolysis; CEA, carcinoembryonic antigen; ROI, region of interest; LASSO, the least absolute shrinkage and selection; ROC, receiver operating curve; AUC, area under the curve; TKI, tyrosine kinase inhibitor; NGTDM, neighborhood gray tone difference matrix; GLRLM, gray level run length matrix; GLCM, gray level co-occurrence matrix; GLSZM, gray level size zone matrix; RS, Rad-score; DT, decision tree; SVM, support vector machine; RF, random forest; GGO, ground-glass opacity.
