# Supplementary material for: PET/CT Radiomic Features: A Potential Biomarker for EGFR Mutation Status and Survival Outcome Prediction in NSCLC Patients Treated With TKIs
Source: Front Oncol. 2022 Jun 21;12:894323. doi: 10.3389/fonc.2022.894323 (PMC9253544; doi:10.3389/fonc.2022.894323)

Supplemental Data

Supplementary Material 1 Radiomics Features

This section contains the definitions of the various features that can be extracted using PyRadiomics. They are subdivided into the following classes:

• First Order Statistics (19 features)

• Shape-based (16 features)

• Gray Level Cooccurence Matrix (24 features)

• Gray Level Run Length Matrix (16 features)

• Gray Level Size Zone Matrix (16 features)

• Neigbouring Gray Tone Difference Matrix (5 features)

• Gray Level Dependence Matrix (14 features)

Specific radiomic features are listed as follows:

• First Order Statistics (19 features)

1. Energy
2. Total Energy
3. Entropy
4. Minimum
5. 10th percentile
6. 90th percentile
7. Maximum
8. Mean
9. Median
10. Interquartile Range
11. Range
12. Mean Absolute Deviation (MAD)
13. Robust Mean Absolute Deviation (rMAD)

14. Root Mean Squared (RMS)

15. AbsoluteDeviation

16. Skewness

17. Kurtosis

18. Variance

19. Uniformity

• Shape-based (14 features)

1. Flatness
2. Least Axis Length
3. Major Axis Length
4. Maximum 2D DiameterColumn
5. Maximum 2D DiameterRow
6. Maximum 2D DiameterSlice
7. Maximum 3D Diameter
8. MeshVolume
9. Minor Axis Length
10. Sphericity
11. SurfaceArea
12. Surface Volume Ratio
13. Voxel Volume
14. Elongation

• Gray Level Cooccurence Matrix (24 features)

1. Autocorrelation
2. joint Average
3. Cluster Prominence
4. Cluster Shade
5. Cluster Tendency
6. Contrast

7. Correlation
8. Difference Entropy
9. Difference Variance
10. Difference Average
11. Joint Energy
12. Joint Entropy
13. Informational Measure of Correlation (IMC) 1
14. Informational Measure of Correlation (IMC) 2
15. Inverse Difference Moment (IDM
16. Maximal Correlation Coefficient (MCC
17. Inverse Difference Moment Normalized (IDMN)
18. Inverse Difference (ID)
19. Inverse Difference Normalized (IDN)
20. Inverse Variance
21. Maximum Probability
22. Sum Average
23. Sum Entropy
24. Sum of Squares

• **Gray Level Run Length Matrix (16 features)**

1. Short Run Emphasis (SRE)
2. Long Run Emphasis (LRE)
3. Gray Level Non-Uniformity (GLN)
4. Gray Level Non-Uniformity Normalized (GLNN)
5. Run Length Non-Uniformity (RLN)
6. Run Length Non-Uniformity Normalized (RLNN)
7. Run Percentage (RP)
8. Gray Level Variance (GLV)
9. Run Variance (RV)

10. Run Entropy (RE)
11. Low Gray Level Run Emphasis (LGLRE)
12. High Gray Level Run Emphasis (HGLRE)
13. Short Run Low Gray Level Emphasis (SRLGLE)
14. Short Run High Gray Level Emphasis (SRHGLE)
15. Long Run Low Gray Level Emphasis (LRLGLE)
16. Long Run High Gray Level Emphasis (LRHGLE)

• Gray Level Size Zone Matrix (16 features)

1. Small Area Emphasis (SAE)
2. Large Area Emphasis (LAE)
3. Gray Level Non-Uniformity (GLN)
4. Gray Level Non-Uniformity Normalized (GLNN)
5. Size-Zone Non-Uniformity (SZN)
6. Size-Zone Non-Uniformity Normalized (SZNN)
7. Zone Percentage (ZP
8. Gray Level Variance (GLV)
9. Zone Variance (ZV)
10. Zone Entropy (ZE)
11. Low Gray Level Zone Emphasis (LGLZE)
12. High Gray Level Zone Emphasis (HGLZE)
13. Small Area Low Gray Level Emphasis (SALGLE)
14. Small Area High Gray Level Emphasis (SAHGLE)
15. Large Area Low Gray Level Emphasis (LALGLE)
16. Large Area High Gray Level Emphasis (LAHGLE)

• Neigbouring Gray Tone Difference Matrix (5 features)

1. Busyness
2. Coarseness
3. Complexity

4. Contrast
5. Strength

• Gray Level Dependence Matrix (14 features)

1. Small Dependence Emphasis (SDE)
2. . Large Dependence Emphasis (LDE)
3. Gray Level Non-Uniformity (GLN)
4. Dependence Non-Uniformity (DN)
5. Dependence Non-Uniformity Normalized (DNN)
6. Gray Level Variance (GLV
7. Dependence Variance (DV)
8. Dependence Entropy (DE)
9. Low Gray Level Emphasis (LGLE)
10. High Gray Level Emphasis (HGLE)
11. Small Dependence Low Gray Level Emphasis (SDLGLE)
12. Small Dependence High Gray Level Emphasis (SDHGLE)
13. Large Dependence Low Gray Level Emphasis (LDLGLE)
14. Large Dependence High Gray Level Emphasis (LDHGLE)

Supplementary Table 1 Univariate logistic regression analysis of clinicopathological factors predicting EGFR mutation status in NSCLC.

Variables	EGFR-WT	EGFR-21-MT			EGFR-19-MT		
		OR	95%CI	*p* value	OR	95%CI	*p* value
**Gender**	Ref.	0.157	（0.088-0.278）	<0.001	0.266	（0.148-0.479）	<0.001
**Smoking**	Ref.	5.167	（1.676-15.926）	0.004	15.500	（4.437-54.143）	<0.001
**Pathological stage**	Ref.	1.078	（0.610-1.097）	0.795	1.085	（0.581-2.026）	0.799
**Location**	Ref.	1.193	（0.632-2.251）	0.586	1.387	（0.721-2.669）	0.327
**Subtype**	Ref.	4.227	（1.680-10.637）	0.102	0.881	（0.219-3.546）	0.858
**CEA_level**	Ref.	0.816	（0.480-1.387）	0.453	0.683	（0.387-1.204）	0.187
**Age**	Ref.	0.983	（0.955-1.013）	0.265	0.974	（0.944-1.005）	0.096
**SUVmax**	Ref.	1.171	（1.115-1.229）	<0.001	1.290	（1.218-1.366）	<0.001
**SUVmean**	Ref.	0.892	（0.809-0.983）	0.021	0.860	（0.770-0.961）	<0.001
**TLG**	Ref.	1.000	（0.999-1.000）	0.461	1.000	（0.999-1.000）	0.672
**Tumor_size**	Ref.	0.895	（0.627-1.277）	0.541	1.364	（0.936-1.989）	0.106

Note: EGFR-WT（EGFR wild type）; EGFR-19-MT (EGFR 19 mutation type); EGFR-21-MT (EGFR 21 mutation type); SUVmax (maximum standardized uptake value); SUV mean (mean standardized uptake value); TLG (total lesion glycolysis); CEA (carcinoembryonic antigen).

Supplementary Table 2 Multivariate logistic regression analysis of clinicopathological factors predicting EGFR mutation status in NSCLC.

Variables	EGFR-WT	EGFR-21-MT			EGFR-19-MT		
		OR	95%CI	*p* value	OR	95%CI	*p* value
**SUVmax**	Ref.	1.186	（1.122-1.253）	<0.001	1.330	（1.241-1.424）	<0.001
**SUVmean**	Ref.	0.966	（0.903-1.033）	0.316	0.745	（0.639-0.868）	0.067
**Gender**	Ref.	0.167	（0.085-0.328）	<0.001	0.287	（0.124-0.664）	<0.001

**Smoking**	Ref.	0.967	（0.475-1.971）	0.927	0.789	（0.320-1.946）	0.607

Note: EGFR-WT（EGFR wild type）; EGFR-19-MT (EGFR 19 mutation type); EGFR-21-MT (EGFR 21 mutation type); SUVmax (maximum standardized uptake value); SUV mean (mean standardized uptake value).

Supplementary Table 3 Radiomics features and corresponding coefficients for predicting EGFR mutation status in NSCLC.

**Feature name**	**Corresponding coefficients**
log-sigma-3-0-mm-3D_glszm_GrayLevelNonUniformityNormalized-CT	5.1472
log.sigma.3.0.mm.3D_ngtdm_Coarseness-CT	1.0255
wavelet-HHH_glcm_Correlation-CT	0.0079
wavelet.LHL_glrlm_GrayLevelNonUniformityNormalized-CT	10.4566
log-sigma-3-0-mm-3D_ngtdm_Busyness-CT	-4.1123
log-sigma-3-0-mm-3D_firstorder_Kurtosis-CT	2.5536
wavelet-LHH_firstorder_Skewness-CT	-0.9128
wavelet-LHL_firstorder_Maximum-CT	4.8829
wavelet-HHL_glcm_ClusterProminence-PET	9.1447
original_glszm_SmallAreaEmphasis-PET	-0.0062
wavelet-HLL_ngtdm_Coarseness-PET	2.1585
wavelet-LHH_glcm_ClusterProminence-PET	7.5240

wavelet-HLH_firstorder_Kurtosis-PET	0.9663
log.sigma.3.0.mm.3D_firstorder_90Percentile-PET	3.2246

The significance of radiomics features is explained as follows:

log-sigma-3-0-mm-3D_glszm_GrayLevelNonUniformityNormalized-CT

Gray Level Non-Uniformity Normalized (GLNN)

$$GLNN = \frac{\sum_{i=1}^{N_g}\left(\sum_{j=1}^{N_s}\mathbf{P}(i,j)\right)^2}{N_z^2}$$

GLNN measures the variability of gray-level intensity values in the image, with a lower value indicating a greater similarity in intensity values. This is the normalized version of the GLN formula.

log.sigma.3.0.mm.3D_ngtdm_Coarseness-CT

Calculate and return the coarseness.

$$Coarseness = \frac{1}{\sum_{i=1}^{N_g} p_i s_i}$$

Coarseness is a measure of average difference between the center voxel and its neighbourhood and is an indication of the spatial rate of change. A higher value indicates a lower spatial change rate and a locally more uniform texture.

N.B. $\sum_{i=1}^{N_g} p_i s_i$ potentially evaluates to 0 (in case of a completely homogeneous image). If this is the case, an arbitrary value of 106 is returned.

wavelet-HHH_glcm_Correlation-CT

$$correlation = \frac{\sum_{i=1}^{N_g}\sum_{j=1}^{N_g} p(i,j)ij - \mu_x\mu_y}{\sigma_x(i)\sigma_y(j)}$$

Correlation is a value between 0 (uncorrelated) and 1 (perfectly correlated) showing the linear dependency of gray level values to their respective voxels in the GLCM.

wavelet.LHL_glrlm_GrayLevelNonUniformityNormalized-CT

Gray Level Non-Uniformity Normalized (GLNN)

$$GLNN = \frac{\sum_{i=1}^{N_g}\left(\sum_{j=1}^{N_r}\mathbf{P}(i,j|\theta)\right)^2}{N_z(\theta)^2}$$

GLNN measures the similarity of gray-level intensity values in the image, where a lower GLNN value correlates with a greater similarity in intensity values. This is the normalized version of the GLN formula

log-sigma-3-0-mm-3D_ngtdm_Busyness-CT

Calculate and return the busyness

$$Busyness = \frac{\sum_{i=1}^{N_g} p_i s_i}{\sum_{i=1}^{N_g} \sum_{j=1}^{N_g} |ip_i - jp_j|}, \text{where } p_i \neq 0, p_j \neq 0$$

A measure of the change from a pixel to its neighbour. A high value for busyness indicates a 'busy' image, with rapid changes of intensity between pixels and its neighbourhood.

N.B. if $N_{g,p} = 1$, then $busyness = \frac{0}{0}$. . If this is the case, 0 is returned, as it concerns a fully homogeneous region.

log-sigma-3-0-mm-3D_firstorder_Kurtosis-CT

$$kurtosis = \frac{\mu_4}{\sigma^4} = \frac{\frac{1}{N_p}\sum_{i=1}^{N_p} (\mathbf{X}(i) - \bar{X})^4}{\left(\frac{1}{N_p}\sum_{i=1}^{N_p} (\mathbf{X}(i) - \bar{X})^2\right)^2}$$

Where μ4 is the 4th central moment.

Kurtosis is a measure of the 'peakedness' of the distribution of values in the image ROI. A higher kurtosis implies that the mass of the distribution is concentrated towards the tail(s) rather than towards the mean. A lower kurtosis implies the reverse: that the mass of the distribution is concentrated towards a spike near the Mean value.

wavelet-LHH_firstorder_Skewness-CT

$$skewness = \frac{\mu_3}{\sigma^3} = \frac{\frac{1}{N_p}\sum_{i=1}^{N_p} (\mathbf{X}(i) - \bar{X})^3}{\left(\sqrt{\frac{1}{N_p}\sum_{i=1}^{N_p} (\mathbf{X}(i) - \bar{X})^2}\right)^3}$$

Where μ3 is the 3rd central moment.

Skewness measures the asymmetry of the distribution of values about the Mean value. Depending on where the tail is elongated and the mass of the distribution is concentrated, this value can be positive or negative.

wavelet-LHL_firstorder_Maximum-CT

maximum = max(X)

The maximum gray level intensity within the ROI.

wavelet-HHL_glcm_ClusterProminence-PET

$$cluster\ prominence = \sum_{i=1}^{N_g} \sum_{j=1}^{N_g} \left(i + j - \mu_x - \mu_y\right)^4 p(i,j)$$

Cluster Prominence is a measure of the skewness and asymmetry of the GLCM. A higher value implies more asymmetry about the mean while a lower value indicates a peak near the mean value and less variation about the mean.

original_glszm_SmallAreaEmphasis-PET

Small Area Emphasis (SAE)

$$SAE = \frac{\sum_{i=1}^{N_g} \sum_{j=1}^{N_s} \frac{\mathbf{P}(i,j)}{j^2}}{N_z}$$

SAE is a measure of the distribution of small size zones, with a greater value indicative of more smaller size zones and more fine textures.

wavelet-HLL_ngtdm_Coarseness-PET

Calculate and return the coarseness.

$$Coarseness = \frac{1}{\sum_{i=1}^{N_g} p_i s_i}$$

Coarseness is a measure of average difference between the center voxel and its neighbourhood and is an indication of the spatial rate of change. A higher value indicates a lower spatial change rate and a locally more uniform texture.

N.B. $\sum_{i=1}^{N_g} p_i s_i$ potentially evaluates to 0 (in case of a completely homogeneous image). If this is the case, an arbitrary value of 10^6 is returned.

wavelet-LHH_glcm_ClusterProminence-PET

$$cluster\ prominence = \sum_{i=1}^{N_g} \sum_{j=1}^{N_g} \left(i + j - \mu_x - \mu_y\right)^4 p(i,j)$$

Cluster Prominence is a measure of the skewness and asymmetry of the GLCM. A higher value implies more asymmetry about the mean while a lower value indicates a peak near the mean value and less variation about the mean.

wavelet-HLH_firstorder_Kurtosis-PET

$$kurtosis = \frac{\mu_4}{\sigma^4} = \frac{\frac{1}{N_p} \sum_{i=1}^{N_p} (\mathbf{X}(i) - \bar{X})^4}{\left(\frac{1}{N_p} \sum_{i=1}^{N_p} (\mathbf{X}(i) - \bar{X})^2\right)^2}$$

Where μ4 is the 4th central moment.

Kurtosis is a measure of the ‘peakedness’ of the distribution of values in the image ROI. A higher kurtosis implies that the mass of the distribution is concentrated towards the tail(s) rather than towards the mean. A lower kurtosis implies the reverse: that the mass of the distribution is concentrated towards a spike near the Mean value.

log.sigma.3.0.mm.3D_firstorder_90Percentile-PET

90th percentile

The 90th percentile of X

Supplementary Table 4 Univariate COX regression analysis of clinicopathological factors predicting OS in NSCLC.

Variables	β	SE	*P*	HR	95%CI

Variables	β	SE	*P*	HR	95%CI
**Mutation site**	−3.363	0.406	<0.001	0.035	(0.016−0.077)
**Gender**	0.133	0.223	0.550	1.143	(0.738−1.769)
**Smoking**	−0.387	0.214	0.071	0.679	(0.446−1.034)
**Pathological stage**	0.464	0.400	0.246	1.590	(0.726−3.479)
**Location**	0.200	0.257	0.437	1.221	(0.738−2.091)
**Subtype**	0.313	0.325	0.336	1.367	(0.723−2.585)
**CEA**	0.143	0.206	<0.001	1.154	(0.771−1.729)
**Age**	−0.001	0.012	0.962	0.999	(0.977−1.023)
**SUVmax**	0.343	0.038	<0.001	1.409	(1.039−1.517)
**SUVmean**	0.064	0.023	<0.001	1.066	(1.020−1.115)
**TLG**	0.262	0.142	0.056	0.669	(0.582−1.019)
**Tumor size**	−0.074	0.135	0.585	0.929	(0.713−1.210)

Note: SUVmax (maximum standardized uptake value); SUV mean (mean standardized uptake value); TLG (total lesion glycolysis); CEA (carcinoembryonic antigen)

Supplementary Table 5 Multivariate COX regression analysis of clinicopathological factors predicting OS in NSCLC

Variables	β	SE	*P*	HR	95%CI
**Gender**	-0.014	0.263	0.958	0.986	(0.589-1.650)
**Age**	0.022	0.014	0.116	1.022	(0.995-1.050)
**Mutation site**	-3.736	0.489	<0.001	0.024	(0.009-0.062)
**SUVmax**	0.191	0.050	<0.001	1.210	(1.097-1.335)
**SUVmean**	-0.005	0.028	0.860	0.995	(0.942-1.051)
**TLG**	0.024	0.014	0.102	1.024	(0.995-1.054)

Variables	β	SE	*P*	HR	95%CI
**CEA**	0.266	0.333	0.425	1.305	(0.679-2.508)

Note: SUVmax (maximum standardized uptake value); SUV mean (mean standardized uptake value); TLG (total lesion glycolysis); CEA (carcinoembryonic antigen)

Supplementary Table 6 Univariate COX regression analysis of clinicopathological factors predicting PFS in NSCLC

Variables	β	SE	*P*	HR	95%CI
**Mutation site**	−3. 390	0. 408	<0.001	0. 034	(0.015-0.075)
**Gender**	-0.035	0. 253	0. 891	0. 966	(0.589-1.584)
**Smoking**	−0. 338	0. 215	0. 115	0. 713	(0.468-1.086)
**Pathological stage**	0. 465	0. 400	0. 245	1. 592	(0.727-3.484)
**Location**	0. 173	0. 257	0. 500	1. 189	(0.719-1.967)
**Subtype**	0. 290	0. 325	0. 371	1. 337	(0.707-2.527)
**CEA**	1. 431	0. 325	<0.001	4. 183	(2.210-7.917)
**Age**	−0. 001	0. 012	0. 909	0. 999	(0.976-1.022)
**SUVmax**	0. 001	0. 000	<0.001	1. 001	(1.000-1.001)
**SUVmean**	0. 076	0. 023	<0.001	1. 079	(1.031-1.129)
**TLG**	−0. 036	0. 013	<0.001	0. 965	(0.941-0.989)
**Tumor size**	−0. 069	0. 132	0. 598	0. 933	(0.720-1.028)

Note: SUVmax (maximum standardized uptake value); SUV mean (mean standardized uptake value); TLG (total lesion glycolysis); CEA (carcinoembryonic antigen)

Supplementary Table 7 Multivariate COX regression analysis of clinicopathological factors predicting PFS in NSCLC

Variables	β	SE	*P*	HR	95%CI
**Mutation site**	−3. 713	0. 478	<0.001	0.026	(0.010-0.062)
**Pathological stage**	0. 634	0. 345	0. 066	1. 885	(0.959-3.706)
**CEA**	0. 034	0. 018	0. 062	1. 035	(0.998-1.073)

Variables	β	SE	*P*	HR	95%CI
**Age**	0. 013	0. 013	0. 329	1. 013	(0.987-1.039)
**SUVmax**	0. 191	0. 050	<0.001	1. 210	(1.097-1.335)
**SUVmean**	0. 018	0. 027	0. 501	1. 019	(0.965-1.075)
**TLG**	−0. 036	0. 013	0. 005	0. 965	(0.941-0.989)

Note: SUVmax (maximum standardized uptake value); SUV mean (mean standardized uptake value); TLG (total lesion glycolysis); CEA (carcinoembryonic antigen)

Supplementary Table 8 The significance of Radiomics features in Radiomics Nomo to OS prediction is explained as follows:

**Feature name**
original_glrlm_LowGrayLevelRunEmphasis.CT
original_glszm_LowGrayLevelZoneEmphasis.CT
wavelet.HHL_glcm_Correlation.CT
wavelet.HLH_glszm_ZonePercentage.CT
wavelet.LHH_gldm_SmallDependenceEmphasis.CT
original_shape_LeastAxisLength.PET
wavelet.LHH_glcm_Idmn.PET
wavelet.LLH_firstorder_Kurtosis.PET

The significance of radiomics features is explained as follows:

original_glrlm_LowGrayLevelRunEmphasis.CT

Low Gray Level Run Emphasis (LGLRE)

$$LGLRE = \frac{\sum_{i=1}^{N_g}\sum_{j=1}^{N_r}\frac{\mathbf{P}(i,j|\theta)}{i^2}}{N_z(\theta)}$$

LGLRE measures the distribution of low gray-level values, with a higher value indicating a greater concentration of low gray-level values in the image.

original_glszm_LowGrayLevelZoneEmphasis.CT

Low Gray Level Zone Emphasis (LGLZE)

$$LGLZE = \frac{\sum_{i=1}^{N_g}\sum_{j=1}^{N_s}\frac{\mathbf{P}(i,j)}{i^2}}{N_z}$$

LGLZE measures the distribution of lower gray-level size zones, with a higher value indicating a greater proportion of lower gray-level values and size zones in the image.

wavelet.HHL_glcm_Correlation.CT

$$correlation = \frac{\sum_{i=1}^{N_g}\sum_{j=1}^{N_g} p(i,j)ij - \mu_x\mu_y}{\sigma_x(i)\sigma_y(j)}$$

Correlation is a value between 0 (uncorrelated) and 1 (perfectly correlated) showing the linear dependency of gray level values to their respective voxels in the GLCM.

wavelet.HLH_glszm_ZonePercentage.CT

Zone Percentage (ZP)

$$ZP = \frac{N_z}{N_p}$$

ZP measures the coarseness of the texture by taking the ratio of number of zones and number of voxels in the ROI.

Values are in range 1 Np ≤ ZP ≤ 1, with higher values indicating a larger portion of the ROI consists of small zones (indicates a finer texture).

wavelet.LHH_gldm_SmallDependenceEmphasis.CT

Small Dependence Emphasis (SDE)

$$SDE = \frac{\sum_{i=1}^{N_g}\sum_{j=1}^{N_d}\frac{\mathbf{P}(i,j)}{i^2}}{N_z}$$

A measure of the distribution of small dependencies, with a greater value indicative of smaller dependence and less homogeneous textures.

original_shape_LeastAxisLength.PET

Least Axis Length

$$least\ axis = 4\sqrt{\lambda_{least}}$$

This feature yields the smallest axis length of the ROI-enclosing ellipsoid and is calculated using the largest principal component λleast. In case of a 2D segmentation, this value will be 0.

The principal component analysis is performed using the physical coordinates of the voxel centers defining the ROI. It therefore takes spacing into account, but does not make use of the shape mesh. **wavelet.LHH_glcm_Idmn.PET**

Inverse Difference Moment Normalized (IDMN)

$$IDMN = \sum_{k=0}^{N_g-1}\frac{p_{x-y}(k)}{1+\left(\frac{k^2}{N_g^2}\right)}$$

IDMN (inverse difference moment normalized) is a measure of the local homogeneity of an image. IDMN weights are the inverse of the Contrast weights (decreasing exponentially from the diagonal i = j in the GLCM). Unlike Homogeneity2, IDMN normalizes the square of the difference between neighboring intensity values by dividing over the square of the total number of discrete intensity values.

wavelet.LLH_firstorder_Kurtosis.PET

$$kurtosis = \frac{\mu_4}{\sigma^4} = \frac{\frac{1}{N_p}\sum_{i=1}^{N_p}(\mathbf{X}(i) - \bar{X})^4}{\left(\frac{1}{N_p}\sum_{i=1}^{N_p}(\mathbf{X}(i) - \bar{X})^2\right)^2}$$

Where μ4 is the 4th central moment.

Kurtosis is a measure of the ‘peakedness’ of the distribution of values in the image ROI. A higher kurtosis implies that the mass of the distribution is concentrated towards the tail(s) rather than towards the mean. A lower kurtosis implies the reverse: that the mass of the distribution is concentrated towards a spike near the Mean value.

Supplementary Table 9 The significance of Radiomics features in Radiomics Nomo to PFS prediction is explained as follows:

**Feature name**
original_glrlm_ShortRunLowGrayLevelEmphasis.CT
original_glszm_LowGrayLevelZoneEmphasis.CT
original_shape_Flatness.CT
wavelet.HHL_glcm_Correlation.CT
wavelet.LLH_glcm_Correlation.CT
original_shape_LeastAxisLength.PET
wavelet.HLL_glcm_Idn.PET
wavelet.LHH_glcm_Idmn.PET
wavelet.LLH_firstorder_Kurtosis.PET

The significance of radiomics features is explained as follows:

original_glrlm_ShortRunLowGrayLevelEmphasis.CT

Short Run Low Gray Level Emphasis (SRLGLE)

$$SRLGLE = \frac{\sum_{i=1}^{N_g}\sum_{j=1}^{N_r}\frac{\mathbf{P}(i,j|\theta)}{i^2 j^2}}{N_z(\theta)}$$

SRLGLE measures the joint distribution of shorter run lengths with lower gray-level values.

original_glszm_LowGrayLevelZoneEmphasis.CT

Low Gray Level Zone Emphasis (LGLZE)

$$LGLZE = \frac{\sum_{i=1}^{N_g}\sum_{j=1}^{N_s}\frac{\mathbf{P}(i,j)}{i^2}}{N_z}$$

LGLZE measures the distribution of lower gray-level size zones, with a higher value indicating a greater proportion of lower gray-level values and size zones in the image.

original_shape_Flatness.CT

Flatness shows the relationship between the largest and smallest principal components in the ROI shape. For computational reasons, this feature is defined as the inverse of true flatness.

$$flatness = \sqrt{\frac{\lambda_{least}}{\lambda_{major}}}$$

Here, λmajor and λleast are the lengths of the largest and smallest principal component axes. The values range between 1 (non-flat, sphere-like) and 0 (a flat object, or single-slice segmentation).

The principal component analysis is performed using the physical coordinates of the voxel centers defining the ROI. It therefore takes spacing into account, but does not make use of the shape mesh. **wavelet.HHL_glcm_Correlation.CT**

$$correlation = \frac{\sum_{i=1}^{N_g}\sum_{j=1}^{N_g} p(i,j)ij - \mu_x\mu_y}{\sigma_x(i)\sigma_y(j)}$$

Correlation is a value between 0 (uncorrelated) and 1 (perfectly correlated) showing the linear dependency of gray level values to their respective voxels in the GLCM. **wavelet.LLH_glcm_Correlation.CT**

$$correlation = \frac{\sum_{i=1}^{N_g}\sum_{j=1}^{N_g} p(i,j)ij - \mu_x\mu_y}{\sigma_x(i)\sigma_y(j)}$$

Correlation is a value between 0 (uncorrelated) and 1 (perfectly correlated) showing the linear dependency of gray level values to their respective voxels in the GLCM.

original_shape_LeastAxisLength.PET

Least Axis Length

$$least\ axis = 4\sqrt{\lambda_{least}}$$

This feature yield the smallest axis length of the ROI-enclosing ellipsoid and is calculated using the largest principal component λleast. In case of a 2D segmentation, this value will be 0.

The principal component analysis is performed using the physical coordinates of the voxel centers defining the ROI. It therefore takes spacing into account, but does not make use of the shape mesh.

wavelet.HLL_glcm_Idn.PET

Inverse Difference Normalized (IDN)

$$IDN = \sum_{k=0}^{N_g-1} \frac{p_{x-y}(k)}{1+\left(\frac{k}{N_g}\right)}$$

IDN (inverse difference normalized) is another measure of the local homogeneity of an image. Unlike Homogeneity1, IDN normalizes the difference between the neighboring intensity values by dividing over the total number of discrete intensity values.

wavelet.LHH_glcm_Idmn.PET

Inverse Difference Moment Normalized (IDMN)

$$IDMN = \sum_{k=0}^{N_g-1} \frac{p_{x-y}(k)}{1+\left(\frac{k^2}{N_g^2}\right)}$$

IDMN (inverse difference moment normalized) is a measure of the local homogeneity of an image. IDMN weights are the inverse of the Contrast weights (decreasing exponentially from the diagonal i = j in the GLCM). Unlike Homogeneity2, IDMN normalizes the square of the difference between neighboring intensity values by dividing over the square of the total number of discrete intensity values.

wavelet.LLH_firstorder_Kurtosis.PET

$$kurtosis = \frac{\mu_4}{\sigma^4} = \frac{\frac{1}{N_p}\sum_{i=1}^{N_p}(\mathbf{X}(i)-\bar{X})^4}{\left(\frac{1}{N_p}\sum_{i=1}^{N_p}(\mathbf{X}(i)-\bar{X})^2\right)^2}$$

Where μ4 is the 4th central moment.

Kurtosis is a measure of the ‘peakedness’ of the distribution of values in the image ROI. A higher kurtosis implies that the mass of the distribution is concentrated towards the tail(s) rather than towards the mean. A lower kurtosis implies the reverse: that the mass of the distribution is concentrated towards a spike near the Mean value.

Supplementary Figure 1 Overall survival (OS) curve according to EGFR mutant subtypes.

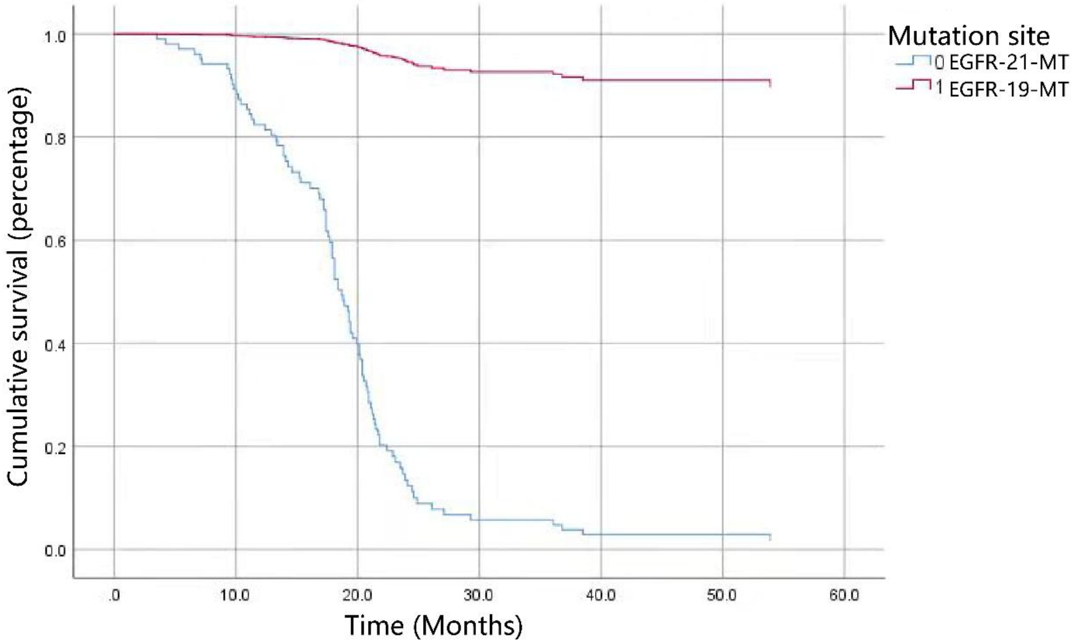

Supplementary Figure 2 Progression-free survival (PFS) curves according to EGFR mutant subtypes

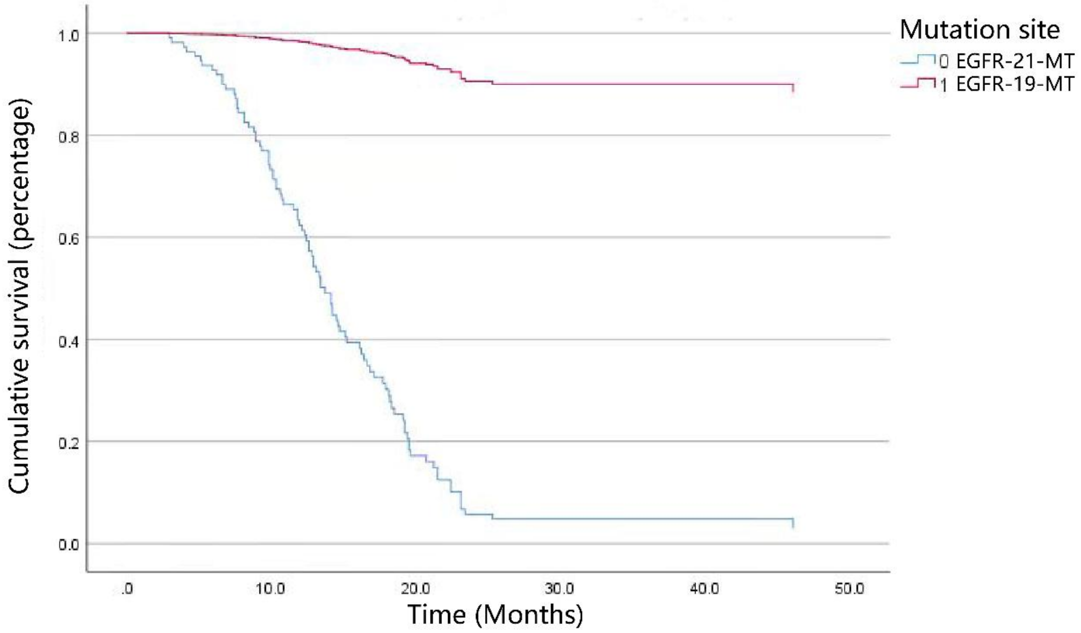

Supplementary Figure 3 Overall survival (OS) curves according to maximum standardized uptake values (SUVmax) values.

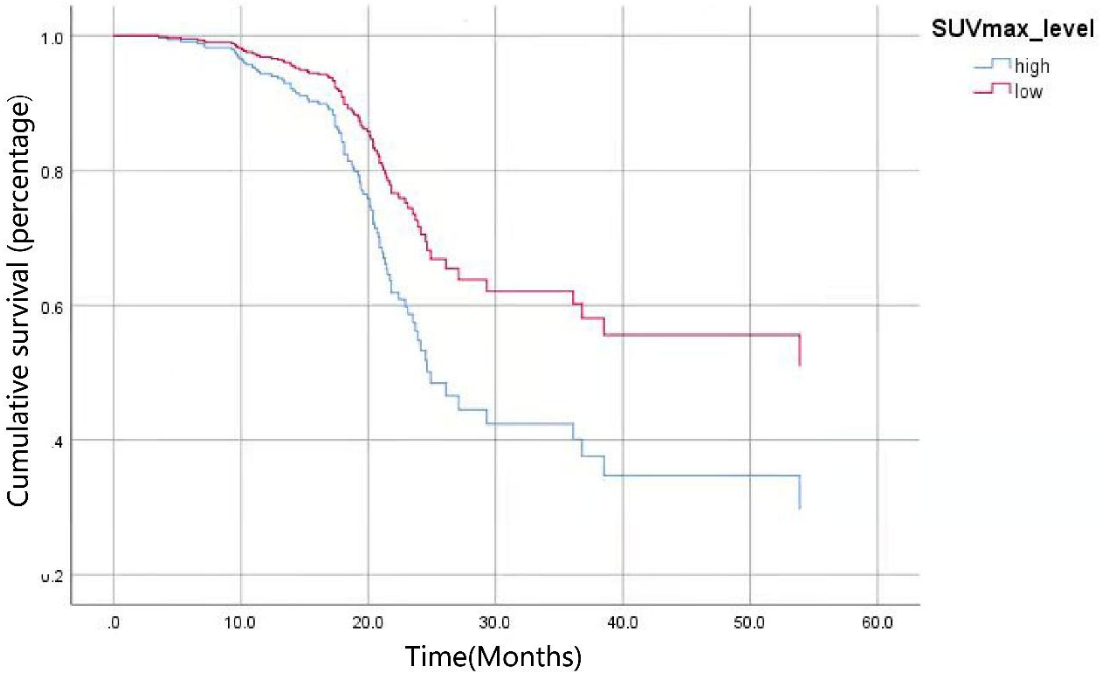

Supplementary Figure 4 Progression-free survival (PFS) curves according to maximum standardized uptake values (SUVmax) values.

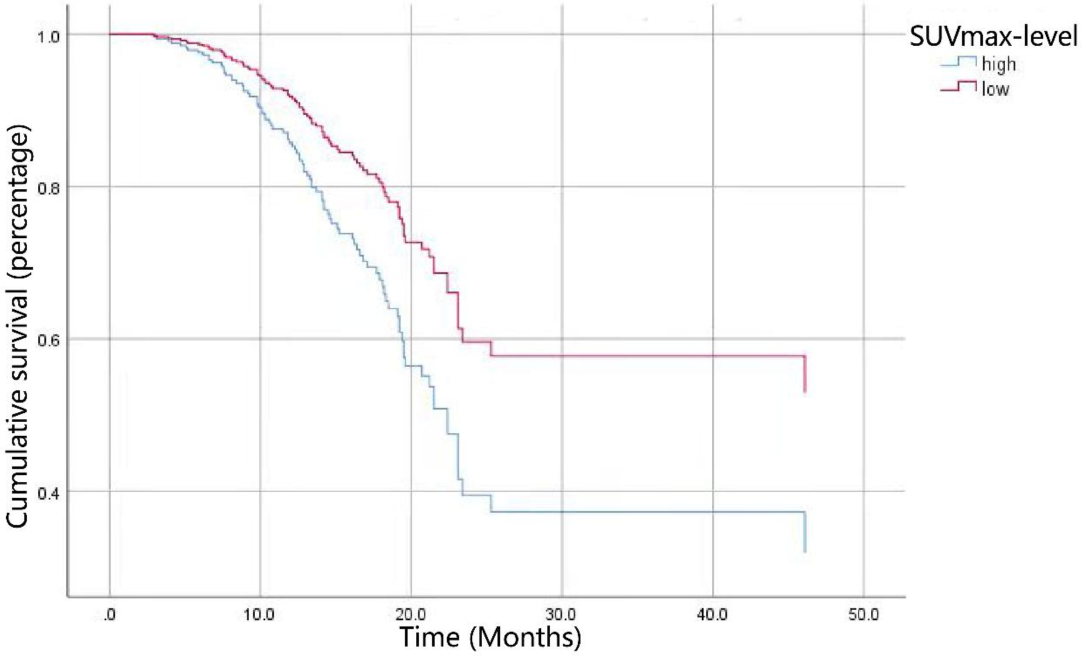

Supplement: Supplementary file 1 [file DataSheet_1.pdf]
